# Longitudinal enlargement of choroid plexus is associated with chronic lesion expansion and neurodegeneration in RRMS patients

**DOI:** 10.1177/13524585241228423

**Published:** 2024-02-06

**Authors:** Samuel Klistorner, Michael H Barnett, Chenyu Wang, John Parratt, Con Yiannikas, Alexander Klistorner

**Affiliations:** Save Sight Institute, Sydney Medical School, The University of Sydney, Sydney, NSW, Australia; Brain and Mind Centre, The University of Sydney, Sydney, NSW, Australia; Sydney Neuroimaging Analysis Centre, Camperdown, NSW, Australia; Royal Prince Alfred Hospital, Sydney, NSW, Australia; Brain and Mind Centre, The University of Sydney, Sydney, NSW, Australia/Sydney Neuroimaging Analysis Centre, Camperdown, NSW, Australia; Royal North Shore Hospital, Sydney, NSW, Australia; Royal North Shore Hospital, Sydney, NSW, Australia; Save Sight Institute, Sydney Medical School, The University of Sydney, Sydney, NSW, Australia

**Keywords:** Atrophy, axonal loss, demyelination, MRI, relapsing/remitting, choroid plexus

## Abstract

**Background and Objective:**

We explored dynamic changes in the choroid plexus (CP) in patients with relapsing-remitting multiple sclerosis (RRMS) and assessed its relationship with chronic lesion expansion and atrophy in various brain compartments.

**Methods:**

Fifty-seven RRMS patients were annually assessed for a minimum of 48 months with 3D FLAIR, pre- and post-contrast 3D T1 and diffusion-weighted magnetic resonance imaging (MRI). The CP was manually segmented at baseline and last follow-up.

**Results:**

The volume of CP significantly increased by 1.4% annually. However, the extent of CP enlargement varied considerably among individuals (ranging from −3.6 to 150.8 mm^3^ or −0.2% to 6.3%). The magnitude of CP enlargement significantly correlated with central (*r* = 0.70, *p* < 0.001) and total brain atrophy (*r* = −0.57, *p* < 0.001), white (*r* = −0.61, *p* < 0.001) and deep grey matter atrophy (*r* = −0.60, *p* < 0.001). Progressive CP enlargement was significantly associated with the volume and extent of chronic lesion expansion (*r* = 0.60, *p* < 0.001), but not with the number or volume of new lesions.

**Conclusion:**

This study provides evidence of progressive CP enlargement in patients with RRMS. Our findings also demonstrate that enlargement of the CP volume is linked to the expansion of chronic lesions and neurodegeneration of periventricular white and grey matter in RRMS patients.

## Introduction

Multiple sclerosis (MS) is a chronic neurodegenerative disease characterized by the presence of inflammatory demyelinating lesions in the central nervous system (CNS). The progression of MS is complex and involves a combination of immune-mediated inflammation and neurodegenerative processes. Acute MS lesion formation is characterized by disruption of the blood–brain barrier (BBB) and infiltration of adaptive immune cells and monocytes. However, concomitant with this acute process, a gradual accumulation of low-grade inflammation occurs within the CNS, referred to as compartmentalized inflammation. This smouldering inflammatory demyelination at the periphery of chronic MS lesions induces lesion expansion over time and is implicated in disease progression, including neurodegeneration, brain atrophy and worsening disability.^1–4^

Recent studies investigated the role of the choroid plexus (CP), a highly vascularized structure within the brain ventricles, in the pathophysiology of MS.^5–11^ CP plays a critical role in regulating the composition of cerebrospinal fluid (CSF) and facilitating the entry of immune cells into the CNS. It has also been implicated in the initiation and propagation of neuroinflammatory processes in MS.

A recent cross-sectional study revealed a correlation between the size of the CP and the degree of chronic lesion expansion^8^ suggesting that alterations in the CP may be linked to the progression of chronic lesions in MS. A separate study focusing on patients with clinically isolated syndrome (CIS) demonstrated a transient increase in the size of the CP during new bouts of acute inflammation.^9^ This suggests a dynamic relationship between the CP and acute inflammatory processes in the CNS.

Given these findings, there is a need to investigate the longitudinal changes in the CP among patients with relapsing-remitting multiple sclerosis (RRMS) and explore their potential association with both acute and chronic inflammation and neurodegeneration.

## Methods

The study was approved by the University of Sydney Human Research Ethics Committees and followed the tenets of the Declaration of Helsinki. Written informed consent was obtained from all participants.

### Subjects

Patients diagnosed with RRMS according to the 2010 or 2017 revised McDonald criteria,^12^ depending on the time of their inclusion, who were enrolled in an on-going longitudinal study of MS-related axonal loss (2014–2022) and completed at least 4 years annual follow-up were included in the study. Patients underwent annual MRI scans and clinical assessment.

### MRI protocol and analysis

MRI was performed using a 3 T GE Discovery MR750 scanner (GE Medical Systems, Milwaukee, WI). The following MRI sequences were acquired annually: Precontrast and postcontrast (gadolinium) sagittal 3D T1, Sagittal 3D T2-FLAIR, diffusion-weighted MRI.

Specific acquisition parameters and MRI image processing are described in more detail in Supplementary Material.

The following brain metrics were analysed using AssemblyNet.^13^: total brain atrophy, white matter atrophy, grey matter atrophy, cortex and deep grey matter atrophy and ventricular volume change.

The degree of tissue damage within lesions and their expanding component was estimated by measuring change in mean diffusivity (MD) between baseline and follow-up timepoints, as previously proposed.^14^

The workflow used for MRI analysis is shown in Supplemental Fig. 1.

#### Longitudinal CP analysis

**Figure 1.**
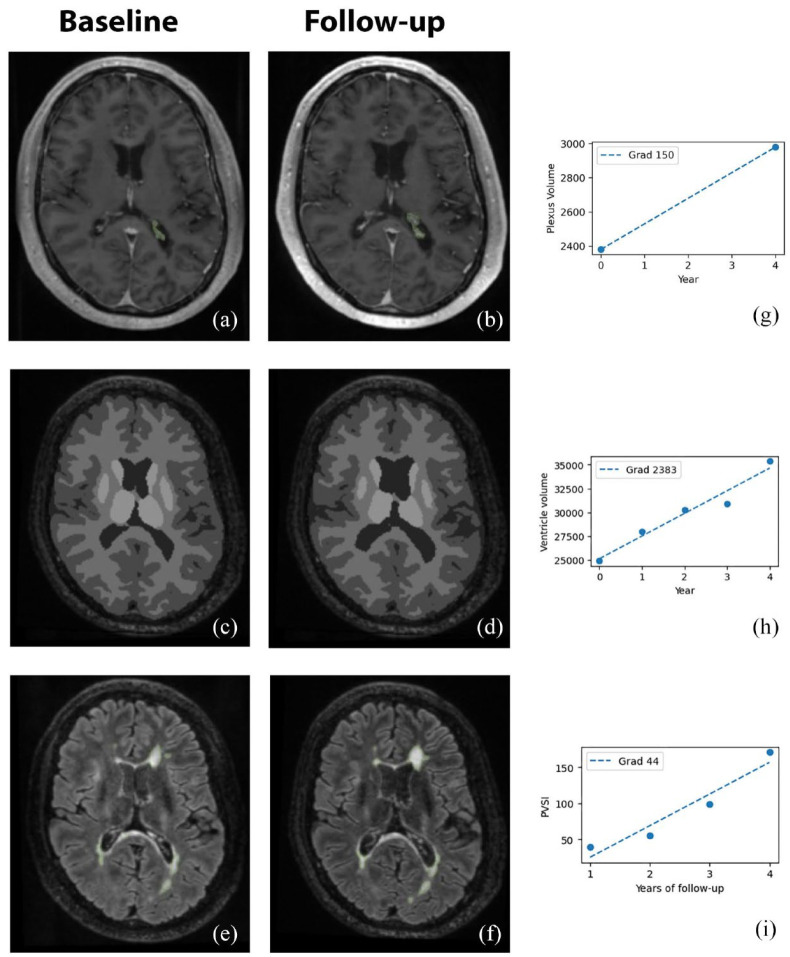
Example of choroid plexus delineation (only right side shown for clarity) using manual segmentation (a, b) (GAD 3D T1), brain tissue segmentation using AssemblyNet (c, d) (3D T1) and lesional segmentation using iQ-MS^TM^ (e, f) (FLAIR). All images presented at baseline and last follow-up. Right column shows values for plexus volume measured at baseline and last follow-up visit (g), ventricle volume values (h) and chronic lesion volume values measured annually (i). The line of best fit represents the annual gradient of change for each of those measures.

The CP within lateral ventricles was manually segmented on ACPC co-registered T1 Gad-enhanced images^8,15^ (Figure 1, upper row) using JIM 9 software (Xinapse Systems, Essex, UK) by a trained analyst (A.K.) at the baseline and last follow-up visit. The analyst was blinded to both clinical and MRI data.

To account for inter-subject variability of head size, the CP volume was normalized (CPn) using SienaX-based scaling, as described previously.^9^ The average annual normalized CPn volume change was calculated for each patient.

Analysis of acute and chronic lesions and volumetric brain analysis described in Supplementary material.

### Statistics

Statistical analysis was performed using SPSS 22.0 (IBM SPSS Statistics for Windows, Version 22.0. Armonk, NY: IBM Corp). Pearson correlation coefficient was used to measure statistical dependence between two numerical variables. For partial correlation, data were adjusted for age, sex, and disease duration. The average annual volume of new lesions was added to partial correlation for analysis of the relationship between CP change and expansion of chronic lesions. *P* values <0.05 were considered statistically significant. Shapiro–Wilk’s test was used to test for normal distribution. When the data violated the assumption of normal distribution, it was transformed using the square root method before performing the correlation.

To assess the significance of longitudinal changes while accounting for variation in the duration of follow-up between patients, a linear mixed-effects model was employed. In this model, the volume measure of interest (CPn, lesions or various metrics of brain atrophy), collected at the two time points (baseline and follow-up) serve as a dependent variable. The fixed effects of the model comprised time (baseline vs follow-up), while controlling for age, sex, and disease duration. The random effects included the ID of individual subjects to account for the repeated measures on the same subjects over time.

## Result

There were 57 patients who satisfied the inclusion criteria. Demographic data are presented in Table 1. A breakdown of the disease-modifying therapies at baseline is shown in Table 1. During the study period, eight patients were maintained on lower efficacy treatment (injectables, such as interferon and glatiramer acetate, teriflunomide and dimethyl-fumarate),^16^ while 27 patients were receiving higher-efficacy drugs (fingolimod, natalizumab and alemtuzumab).^16^ Three patients were treatment-free, while 19 patients changed treatment category between baseline and follow-up visits.

**Table 1.** Demographic data.

Demographic variable	
Total number of patients	57
Age at enrolment, years	40.4 ± 9.0
Sex	22 M/35 F
Disease duration at enrolment, years	6.9 ± 5.9
Duration of the MRI follow-up, months	69.1 ± 15.4 months
EDSS, median (range)	1.1 (0–3.0)
Treatment at baseline:	
Nil	5
Injectables	15
Glatiramer	7
Dimethyl fumarate	2
Fingolimod	17
Natalizumab	8
Teriflunomide	2
Alemtuzumab	1

MRI: magnetic resonance imaging; EDSS: Expanded Disability Status Scale.

**Figure 2.**
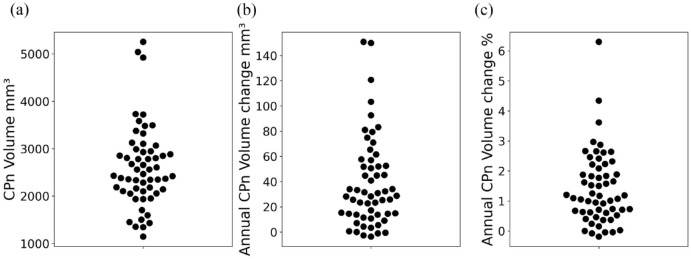
Distribution of CPn volume at baseline (a) and its absolute (b) and relative (c) annual increase.

In our cohort, the average baseline CPn volume was 2616 ± 846 mm^3^ (Figure 2(a)). Over the study period, the volume of CPn increased significantly (*p* < 0.00001, mixed-effect model). The average annual CPn volume enlargement was 38.2 ± 35.6 mm^3^ or 1.4% ± 1.2%. However, the extent of enlargement varied considerably among individuals, ranging from −3.6 to 150.8 mm^3^ or −0.2% to 6.3% (Figure 2(b, c)).

**Figure 3.**
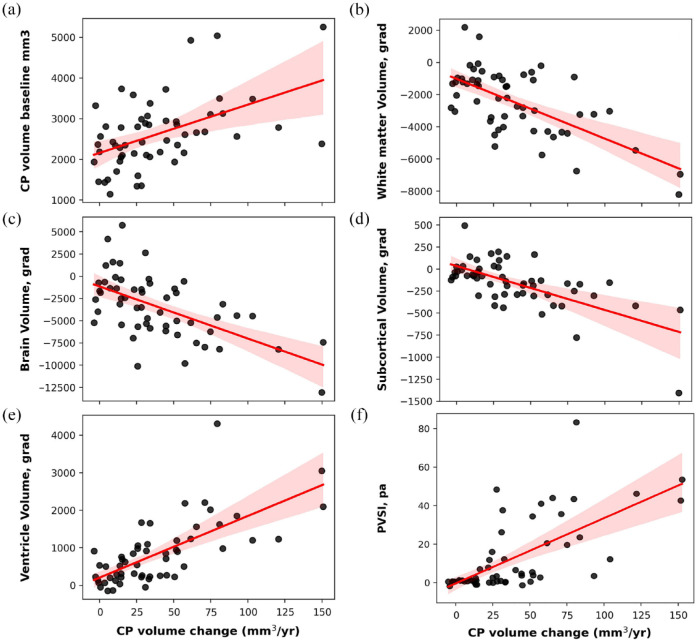
Correlations between longitudinal CPn volume change and CPn volume at baseline (a) and longitudinal brain volumetrics: (b) total brain atrophy, (c) central brain atrophy, (d) white matter atrophy, (e) deep grey matter atrophy and (f) PVSI of chronic lesion expansion. Shaded area represents confidence interval. Gradient (grad) indicates annual change.

The volume of CPn enlargement during follow-up correlated significantly with the size of the CP at baseline (*r* = 0.50, *p* < 0.001; Figure 3(a)), but showed no association with sex, age, disease duration, baseline ventricle volume or Expanded Disability Status Scale (EDSS).

Over the observation period, there was statistically significant total brain atrophy (–0.23% per year), including white matter (–0.48% per year), total grey matter (–0.12% per year), deep grey matter (–0.29% per year) and cortex (–0.11% per year). However, the observed central brain atrophy, as measured by change in ventricular volume, was notably larger (–2.68% per year) (*p* < 0.001 for all, mixed-effect model). Disease duration had a significant effect on total brain atrophy (*p* = 0.001), ventricular volume (*p* = 0.006), white and deep grey matter atrophy (*p* < 0.001 and 0.01 respectively), while age contributed to total brain atrophy (*p* = 0.037), grey matter atrophy (*p* = 0.002) and cortical change (*p* = 0.001).

We also found a substantial increase in total T2 lesion load during the study period (4621 ± 4952and 6076 ± 5981 mm^3^ at baseline and last follow-up, respectively, *p* < 0.0001, mixed-effect model). Duration of follow-up contributed significantly to the model, *p* < 0.001.

There were on average 0.3 ± 0.5 new lesions identified annually (average annual volume of new lesions 45.2 ± 90.0 mm^3^).

Furthermore, we observed significant (*p* < 0.001) expansion of chronic lesions (346 ± 449 mm^3^ average per year), which demonstrated considerable inter-subject variability (coefficient of variability 130%).

Baseline values and annualized change for brain volumetric data are presented in Table 2.

**Table 2.** Brain volumetric data and annual change.

	Total brain volume (mm^3^)	White matter volume (mm^3^)	Grey matter volume (mm^3^)	Cortical volume (mm^3^)	Deep grey matter volume (mm^3^)	Ventricle volume (mm^3^)	CPn (mm^3^)
Baseline	1,577,697 ±51,330	533,337 ±33,353	842,075 ±25,373	784,786 ±24,026	57,289 ±3854	31,640 ±14,435	2116 ± 846
Annual change	−3599 ±3741	2522 ±2066	1046 ±1978	883 ±1860	163 ±270	877 ±880	38.2 ± 35.6

### Association of CPn change with longitudinal brain volumetric parameters

A significant correlation was observed between the annual CPn volume increase and the annual rate of total brain atrophy (*r* = −0.55, *p* < 0.001, partial correlation: *r* = −0.57, *p* < 0.001) (Figure 3(b)). This association noticeably strengthened when atrophy of the central brain (as measured by ventricle enlargement) was considered in isolation (*r* = 0.70, *p* < 0.001, partial correlation: *r* = 0.70, *p* < 0.001) (Figure 3(c)).

CPn enlargement also exhibited a moderate correlation with the annual rate of white matter atrophy (*r* = −0.61, *p* < 0.001, partial correlation: *r* = −0.61, *p* < 0.001) (Figure 3(d)). However, the link between CPn enlargement and grey matter atrophy was weak (*r* = −0.3, *p* = 0.03, partial correlation: *r* = −0.32, *p* = 0.01); and was primarily driven by deep grey matter atrophy (*r* = −0.6, *p* < 0.001, partial correlation: *r* = −0.60, *p* < 0.001) (Figure 3(e)), while the association with cortical grey matter demonstrated borderline significance (*r* = −0.22, *p* = 0.06, partial correlation: *r* = −0.24, *p* = 0.08).

### Association of CPn change with lesions

The annual volume of CPn enlargement significantly correlated with the volume of chronic lesion expansion (*r* = 0.46, *p* < 0.001, partial correlation adjusted for age, sex, disease duration and annual volume of new lesions: *r* = 0.47, *p* < 0.001). The strength of this association markedly increased when the degree of tissue damage within expanding part of the lesion, in the form of the progressive volume/severity index (PVSI),^17^ was considered (*r* = 0.62, *p* < 0.001, partial correlation: *r* = 0.60, *p* < 0.001) (Figure 3(f)). CP enlargement was also linked to the degree of tissue rarefication inside chronic lesions, as measured by MD increase in lesion core (*r* = 0.49, *p* < 0.001).

No significant relationship was found between changes in CP and the number or volume of new lesions.

## Discussion

This study investigated progressive volume change in the CP of patients with RRMS and its associations with biomarkers of both acute and chronic inflammation, as well as brain atrophy.

We established that the volume of the CP in patients with RRMS increases gradually over the course of the disease. We found that the average annual rate of CP enlargement was 1.4%, though there was a wide individual range of change. This is in line with two recently published longitudinal investigations of CP in MS patients.^18,19^

There are several aspects of the study’s design that enabled us to detect such a subtle change. The utilization of manual expert segmentation, despite being a laborious and very time-consuming process, was critical in achieving high-quality delineation of the CP. In addition, extended follow-up of up to 8 years contributed to the robustness of our findings. Namely, the lengthy observation period enabled more accurate estimation of CP change by effectively minimizing measurement error. As a result, it contrasts with our previous study,^8^ in which we were unable to detect changes in CP during a 4-year observation period, thereby indicating the importance of long-term observation in capturing slow and gradual CP volume changes in RRMS.

### Increase of CP volume and inflammation

The vital role of the CP in maintaining the blood–CSF barrier and modulation of inflammatory cells trafficking into the CNS has recently attracted the attention of MS researchers.^20,21^ It has become clear that the CP not only actively participates in acute inflammatory processes including antigen presentation and recruitment of peripheral inflammatory cells,^22^ but remains chronically inflamed, even in long-standing MS,^23^ contributing, therefore, to a proinflammatory state of the CSF and inducing a persistent neuroinflammatory environment in periventricular brain tissue.^24^ This can be further exacerbated by recently demonstrated link between CP enlargement and low periventricular remyelination.^25^

Several recent cross-sectional studies have demonstrated significant enlargement of the CP in patients with MS compared to healthy controls, even at early stages of the disease.6–10,^20^ Various pathomechanisms of CP enlargement in MS have been proposed, including CSF hypersecretion,^26^ oxidative stress,^27^ and edema.^28^ However, numerous experimental, post-mortem and imaging studies have consistently demonstrated the presence of parenchymal inflammation in CP tissue.^5–7,15,23,29^ These studies have also highlighted the close association between this inflammation and the observed increase in CP volume, strongly suggesting that inflammation is the probable cause of this enlargement. Therefore, it is possible that the broad range of CP enlargement observed in our study likely reflects varying degrees of CP inflammation in our RRMS cohort.

This notion aligns with another significant finding of this study – the observed link between progressive CP enlargement and the volume and severity of chronic lesion expansion. This association suggests that CP could be indirectly implicated in forming or sustaining the smouldering inflammatory process at the rim of chronic periventricular MS lesions, possibly through the secretion of pro-inflammatory cytokines or the regulation of immune cell trafficking and microglial activation^30–32^ This view is supported by our earlier study which demonstrated a clear link between the size of the CP and the rate of chronic lesion expansion in patients with RRMS^8^ and also aligns with a recent publication demonstrating larger CP volume in patients with chronic active lesions.^29,33^

Moreover, the link between change of CPn volume and expansion of chronic lesions (which occurs predominantly near the ventricles^34^) may partially explain the predominantly periventricular effect of CP enlargement on brain atrophy. While our results show that CP enlargement significantly correlates with atrophy of both white and grey matter, supporting the view that inflammation originating in the CP may contribute to ongoing neurodegeneration, this association is considerably stronger for the central brain (ventricle enlargement *vs* total brain atrophy or deep grey matter loss *vs* cortical atrophy). This suggests that these relationships are largely driven by the periventricular region, which also demonstrates markedly faster rate of both white and grey matter atrophy compared to the brain periphery.

Furthermore, the significant association of CP enlargement with progressive tissue rarefication inside chronic lesions, as measured by the increase of MD, also strengthens the link between change in CP volume and tissue damage related to axonal transection at the lesion rim.^4,34^

Given the close proximity of subcortical nuclei to the ventricles (and therefore, to the CP), the observed deep grey matter damage might originate from direct influence of CP-induced inflammation or, alternatively, from axonal transection taking place within the rim of chronic expanding lesions that ultimately results in neuronal death. The latter may be more plausible since, in contrast with the deep grey matter, we found no association between CP enlargement and cortical volume. This finding is corroborated by recent research from Wang et al.,^29^ who also established an association between enlarged CP and reduced deep grey matter volume, but found no such relationship with cortical volume.

Notably, we did not find a significant relationship between CP enlargement and the number or volume of new lesions. While suggesting that the CP may relate to, or potentially drive, chronic, rather than the acute, MS pathology, an earlier study revealed a modest, yet significant, correlation between CP size and new lesional activity,^6^ emphasizing the need for further investigation.

Taken together, our study establishes a link between longitudinal enlargement of the CP and various measures of neurodegeneration, including axonal loss in the rim and core of chronic MS lesions, as well as periventricular brain atrophy. Remarkably, while axonal damage related to chronic lesions and the atrophy of the brain’s white and grey matter were derived using different image analysis techniques, they showed similar associations with CP enlargement, increasing robustness of our findings.

There are several limitations to this study. The main limitation is the absence of healthy controls data, which raises the possibility that the observed increase in CP volume might be attributable to age-related changes rather than being specific to MS. However, recently published cross-sectional study by Alisch et al.^35^ suggests that CP volume remains stable in the age range of 20–50 years, which corresponds to the primary age group in our study. This, combined with the lack of association between CP volume enlargement and age in our cohort, as well as the observed significant correlations of CP volume with markers of brain neurodegeneration (which was adjusted for age), supports our hypothesis that observed CP changes may be disease-specific rather than due to normal aging.

Furthermore, while it may be argued that CP enlargement may potentially be caused by increased leakage of contrast through Blood-CSF Barrier, a recent study of contrast circulation in CSF did not find distinct contrast enhancement in the lateral ventricular CSF surrounding the choroid CP, arguing against a possibility that gadolinium leakage may increase visible CP size.^36^ Furthermore, capillary permeability and leakage of contrast in both BBB and BCSFB tend to reduce with age and duration of the MS (due to the overall inflammatory activity decrease), making this factor even less likely to contribute to observed increase of CP volume.^37^

Another limitation is related to the fact that our analysis was based on a relatively small sample of patients with RRMS, which limits the generalizability of the findings. This limitation is particularly relevant in conditions like MS, where there is notable variability in disease progression and response to treatment. Furthermore, the fact that a considerable portion of participants changed treatments during the follow-up period adds a layer of complexity to the data analysis, making it challenging to isolate the effect of any one medication on the longitudinal changes. Due to these factors, our study could not feasibly estimate the potential effects of various treatments on the CP’s longitudinal changes. This is particularly important considering a recent report showing that, contrary to untreated patients or patients treated with dimethyl fumarate, who showed progressive CP enlargement, patients under natalizumab therapy demonstrate stable CP volumes during follow-up.^5^

Moreover, this study was not designed to directly measure inflammation within the CP or brain tissue, and we used volume changes and lesion dynamics as indirect indicators of these processes. More direct measures of inflammation, such as the use of positron emission tomography (PET) with tracers targeting activated microglia or other inflammation-specific markers,^6^ may provide a more comprehensive understanding of the relationship between CP changes and inflammation.

In addition, our study, while longitudinal, was observational in nature. As a result, while we can draw associations from the data, we cannot make causal inferences. It is not clear from our study whether CP enlargement directly contributes to inflammation at the rim of chronic lesions and brain atrophy, or whether they simply occur concurrently due to a shared underlying mechanism.

Finally, while we used manual segmentation for a more accurate estimation of CP volume, this process has its own limitations, including being labour-intensive and subject to human error. Furthermore, it is not feasible for routine clinical use or large-scale studies. The development of automated or semi-automated segmentation methods for CP could potentially overcome this limitation, enabling more widespread and efficient analysis of CP changes in patients with RRMS.^19,38,39^

In conclusion, this study provides evidence of progressive CP enlargement in patients with RRMS and its association with chronic lesion expansion and brain atrophy, supporting a potential role for the CP in the chronic inflammatory processes and neurodegeneration in RRMS.

## Supplemental Material

sj-docx-1-msj-10.1177_13524585241228423 – Supplemental material for Longitudinal enlargement of choroid plexus is associated with chronic lesion expansion and neurodegeneration in RRMS patients Supplemental material, sj-docx-1-msj-10.1177_13524585241228423 for Longitudinal enlargement of choroid plexus is associated with chronic lesion expansion and neurodegeneration in RRMS patients by Samuel Klistorner, Michael H Barnett, Chenyu Wang, John Parratt, Con Yiannikas and Alexander Klistorner in Multiple Sclerosis Journal

## References

[1] Calvi A Carrasco FP Tur C , et al. Association of slowly expanding lesions on MRI with disability in people with secondary progressive multiple sclerosis. Neurology 2022; 98: e1783–e1793. 10.1212/WNL.0000000000200144 35277438

[2] Dal-Bianco A Grabner G Kronnerwetter C , et al. Slow expansion of multiple sclerosis iron rim lesions : Pathology and 7 T magnetic resonance imaging. Acta Neuropathol 2017; 133(1): 25–42. 27796537 10.1007/s00401-016-1636-z PMC5209400

[3] Elliott C Belachew S Wolinsky JS , et al. Chronic white matter lesion activity predicts clinical progression in primary progressive multiple sclerosis. Brain 2019; 142(9): 2787–2799. 31497864 10.1093/brain/awz212 PMC6736181

[4] Klistorner S Barnett MH Yiannikas C , et al. Expansion of chronic lesions is linked to disease progression in relapsing–remitting multiple sclerosis patients. Mult Scler 2021; 27(10): 1533–1542. 33215557 10.1177/1352458520974357

[5] Fleischer V Gonzalez-Escamilla G Ciolac D , et al. Translational value of choroid plexus imaging for tracking neuroinflammation in mice and humans. Proc Natl Acad Sci USA 2021; 118(36): 1–12. 10.1073/pnas.2025000118 PMC8433504 34479997

[6] Ricigliano VAG E Morena E Colombi A , et al. Choroid plexus enlargement in inflammatory multiple sclerosis. Radiology 2021; 301(1): 166–177. 34254858 10.1148/radiol.2021204426

[7] Manouchehri N Stüve O . Choroid plexus volumetrics and brain inflammation in multiple sclerosis. Proc Natl Acad Sci USA 2021; 118(40): 10–12. 10.1073/pnas.2115221118 PMC8501877 34583997

[8] Klistorner S Barnett M Parratt J , et al. Choroid plexus volume predicts expansion of chronic lesions and associated brain atrophy in multiple sclerosis. Ann Clin Transl Neurol 2022; 9: 1528–1537. 36056634 10.1002/acn3.51644 PMC9539382

[9] Klistorner S Van der Walt A Barnett MH , et al. Choroid plexus volume is enlarged in clinically isolated syndrome patients with optic neuritis. Mult Scler 2022; 29: 540–548. 10.1177/13524585231157206 36876595

[10] Ricigliano VAG Louapre C Poirion E , et al. Imaging characteristics of choroid plexuses in presymptomatic multiple sclerosis: A retrospective study. Neurol Neuroimmunol Neuroinflamm 2022; 9(6): e200026. 10.1212/NXI.0000000000200026 PMC9562043 36229188

[11] Müller J Sinnecker T Wendebourg MJ , et al. Choroid plexus volume in multiple sclerosis vs neuromyelitis optica spectrum disorder. Neurol Neuroimmunol Neuroinflamm 2022; 9(3): e1147. 10.1212/NXI.0000000000001147 PMC8883575 35217580

[12] Thompson AJ Banwell B Barkhof F , et al. Diagnosis of multiple sclerosis: 2017 revisions of the McDonald criteria. Lancet Neurol 2018; 17: 162–173. 29275977 10.1016/S1474-4422(17)30470-2

[13] Coupé P Mansencal B Clément M , et al. AssemblyNet: A large ensemble of CNNs for 3D whole brain MRI segmentation. NeuroImage 2020; 219: 117–126. 10.1016/j.neuroimage.2020.117026 32522665

[14] Klistorner A Wang C Yiannikas C , et al. Evidence of progressive tissue loss in the core of chronic MS lesions: A longitudinal DTI study. NeuroImage Clin 2018; 17: 1028–1035. 29387524 10.1016/j.nicl.2017.12.010 PMC5772506

[15] Muthuraman M Oshaghi M Fleischer V , et al. Choroid plexus imaging to track neuroinflammation-a translational model for mouse and human studies. Neural Regen Res 2023; 18(3): 521–522. 36018158 10.4103/1673-5374.346471 PMC9727450

[16] Samjoo IA Worthington E Drudge C , et al. Efficacy classification of modern therapies in multiple sclerosis. J Comp Eff Res 2021; 10(6): 495–507. 33620251 10.2217/cer-2020-0267

[17] Klistorner S Barnett MH Klistorner A . Mechanisms of central brain atrophy in multiple sclerosis. Mult Scler 2022; 28(13): 2038–2045. 35861244 10.1177/13524585221111684

[18] Jankowska A Chwojnicki K Grzywińska M , et al. Choroid plexus volume change—A candidate for a new radiological marker of MS progression. Diagnostics 2023; 13(16): 2668. 37627928 10.3390/diagnostics13162668 PMC10453931

[19] Bergsland N Dwyer MG Jakimovski D , et al. Association of choroid plexus inflammation on MRI with clinical disability progression over 5 years in patients with multiple sclerosis. Neurology 2023; 100(9): E911–E920. 10.1212/WNL.0000000000201608 PMC9990433 36543575

[20] Rodríguez-Lorenzo S Konings J Van Der Pol S , et al. Inflammation of the choroid plexus in progressive multiple sclerosis: Accumulation of granulocytes and T cells. Acta Neuropathol Commun 2020; 8(1): 1–13. 32014066 10.1186/s40478-020-0885-1 PMC6998074

[21] Rodríguez-Lorenzo S Ferreira Francisco DM Vos R , et al. Altered secretory and neuroprotective function of the choroid plexus in progressive multiple sclerosis. Acta Neuropathol Commun 2020; 8(1): 1–13. 32192527 10.1186/s40478-020-00903-y PMC7083003

[22] Dixon GA Pérez CA . Multiple sclerosis and the choroid plexus: Emerging concepts of disease immunopathophysiology. Pediatr Neurol 2020; 103: 65–75. 31780202 10.1016/j.pediatrneurol.2019.08.007

[23] Vercellino M Votta B Condello C , et al. Involvement of the choroid plexus in multiple sclerosis autoimmune inflammation: A neuropathological study. J Neuroimmunol 2008; 199(1–2): 133–141. 18539342 10.1016/j.jneuroim.2008.04.035

[24] Tonietto M Poirion E Lazzarotto A , et al. Periventricular remyelination failure in multiple sclerosis: A substrate for neurodegeneration. Brain 2023; 146(1): 182–194. 36097347 10.1093/brain/awac334

[25] Ricigliano VAG Stankoff B . Choroid plexuses at the interface of peripheral immunity and tissue repair in multiple sclerosis. Curr Opin Neurol 2023; 36(3): 214–221. 37078651 10.1097/WCO.0000000000001160

[26] Barkho BZ Monuki ES . Proliferation of cultured mouse choroid plexus epithelial cells. PLoS ONE 2015; 10(3): e0121738. 10.1371/journal.pone.0121738 PMC4376882 25815836

[27] Campbell G Kraytsberg E Krishnan K , et al. Clonal expansion of mitochondrial DNA deletions in multiple sclerosis. Acta Neuropathol 2012; 124(1): 209–220. 22688405 10.1007/s00401-012-1001-9 PMC3674417

[28] Cardia E Molina D Abbate F , et al. Morphological modifications of the choroid plexus in a rodent model of acute ventriculitis induced by gram-negative liquoral sepsis – Possible implications in the pathophysiology of hypersecretory hydrocephalus. Childs Nerv Syst 1995; 11(9): 511–516. 8529217 10.1007/BF00822840

[29] Wang X Zhu Q Yan Z , et al. Enlarged choroid plexus related to iron rim lesions and deep gray matter atrophy in relapsing-remitting multiple sclerosis. Mult Scler Relat Disord 2023; 75: 104740. 37146422 10.1016/j.msard.2023.104740

[30] Poirion E Tonietto M Lejeune FX , et al. Structural and clinical correlates of a periventricular gradient of neuroinflammation in multiple sclerosis. Neurology 2021; 96(14): e1865–e1875. 10.1212/WNL.0000000000011700 PMC8105971 33737372

[31] Magliozzi R Howell OW Reeves C , et al. A gradient of neuronal loss and meningeal inflammation in multiple sclerosis. Ann Neurol 2010; 68(4): 477–493. 20976767 10.1002/ana.22230

[32] Magliozzi R Scalfari A Pisani AI , et al. The CSF profile linked to cortical damage predicts multiple sclerosis activity. Ann Neurol 2020; 88(3): 562–573. 32418239 10.1002/ana.25786

[33] Hamzaoui M Garcia J Boffa G , et al. Positron emission tomography with [18F]-DPA-714 unveils a smoldering component in most multiple sclerosis lesions which drives disease progression. Ann Neurol 2023; 94(2): 366–383. 37039158 10.1002/ana.26657

[34] Klistorner S Barnett MH Graham SL , et al. The expansion and severity of chronic MS lesions follows a periventricular gradient. Mult Scler 2022; 28(10): 1504–1514. 35296170 10.1177/13524585221080667

[35] Alisch JSR Kiely M Triebswetter C , et al. Characterization of age-related differences in the human choroid plexus volume, microstructural integrity, and blood perfusion using multiparameter magnetic resonance imaging. Front Aging Neurosci 2021; 1313: 734992. 10.3389/fnagi.2021.734992 PMC8485051 34603011

[36] Ohashi T Naganawa S Iwata S , et al. Distribution of gadolinium-based contrast agent after leaking into the cerebrospinal fluid: Comparison between the cerebral cisterns and the lateral ventricles. Magn Reson Med Sci 2021; 20(2): 175–181. 32641590 10.2463/mrms.mp.2020-0016 PMC8203476

[37] Bouzerar R Chaarani B Gondry-Jouet C , et al. Measurement of choroid plexus perfusion using dynamic susceptibility MR imaging: Capillary permeability and age-related changes. Neuroradiology 2013; 55(12): 1447–1454. 24150596 10.1007/s00234-013-1290-2

[38] Yazdan-Panah A Schmidt-Mengin M Ricigliano VAG , et al. Automatic segmentation of the choroid plexuses: Method and validation in controls and patients with multiple sclerosis. NeuroImage Clin 2023; 38: 103368. 36913908 10.1016/j.nicl.2023.103368 PMC10011049

[39] Storelli L Pagani E Rubin M , et al. A fully automatic method to segment choroid plexuses in multiple sclerosis using conventional MRI sequences. J Magn Reson Imaging. Epub ahead of print 2 August 2023. DOI: 10.1002/jmri.28937. 37530734

