# Supplementary material for: Longitudinal enlargement of choroid plexus is associated with chronic lesion expansion and neurodegeneration in RRMS patients
Source: Mult Scler. 2024 Feb 6;30(4-5):496–504. doi: 10.1177/13524585241228423 (PMC11010552; doi:10.1177/13524585241228423)

1. **Specific MRI parameters and image processing.**

The following MRI sequences were acquired:

1. Pre- and post-contrast (gadolinium) Sagittal 3D T1: GE BRAVO sequence, duration 4 min each, FOV 256mm, Slice thickness 1mm, TE 2.7ms, TR 7.2ms, Flip angle 12°, Pixel spacing 1mm. Acquisition Matrix (Freq x Phase) is 256x256, which results in 1mm isotropic acquisition voxel size. The reconstruction matrix is 256x256.
2. FLAIR CUBE; GE CUBE T2 FLAIR sequence, duration 6 min, FOV 240mm, Slice thickness 1.2 mm, Acquisition Matrix (Freq x Phase) 256x244, TE 163ms, TR 8000ms, Flip angle 90°, Pixel spacing 0.47 mm. The reconstruction matrix is 512x512.
3. Echo-Planar Imaging based diffusion weighted MRI, duration 9 min (64-directions with 2 mm isotropic acquisition matrix, TR/TE = 8325/86 ms, b = 1000 s/mm^2^, number of b0 = 2).

**MRI image pre-processing:**

**﻿** The baseline T1-weighted imaging was realigned to Anterior and Posterior Commissure (AC-PC) orientation. Using FLIRT (FSL, FMRIB Software Library), follow-up T1 images were co-registered to initial (month 0) AC-PC space by applying transformation matrices derived from linear co-registration between baseline AC-PC aligned brain and follow-up native T1 brain images. In parallel, diffusion MRI was corrected for motion and eddy-current distortion in FSL, then EPI susceptibility distortion was minimized by applying deformation maps generated from nonlinear co-registration between DWI b0 brain images and T1-weighted images at each time-point using ANTs (Advanced Normalization Tools). Subsequently, tensor reconstruction was performed in MRtrix3. Tensor and FLAIR images were then linearly co-registered to corresponding T1 AC-PC images at each timepoint.

**DTI data processing:**

Diffusion weighted MRI data (dMRI) were pre-processed using tools provided by the software suites MRtrix3, FSL, and ANTs. Specifically, dMRI data were first denoised, then potential Gibbs-ringing artefacts were removed. The dMRI data were then corrected for bias field inhomogeneities using the ANTs N4 algorithm. a dMRI brain mask was then estimated using BET and used as an input, alongside the dMRI data, to eddy in order to correct for subject movement in the acquisition. Finally, phase distortion correction was applied using a non-linear registration method outlined below.

To correct for phase distortion within the dMRI data, first the brain was segmented from the corresponding T1w dataset, and a single b0 volume was extracted from the dMRI dataset. The T1w brain was then used as a mask to invert the contrast of the T1w image. A rigid-body registration was then performed on the inverted T1w image with the b0 volume as a target, which aligned the two images spatially. Non-linear registration was then performed using ANTs. The registration steps were comprised of a rigid body, then affine, and then SyN registration algorithm. The transformations and warps calculated from the non-linear registration steps were then applied to the entire dMRI dataset to correct for phase distortion artefacts.

**Analysis of chronic lesions:**

T2 lesions were segmented for all time points employing a fully automated lesion segmentation algorithm from the iQ-MS^TM^ software suite (Sydney Neuroimaging Analysis Centre, Sydney, Australia), using unprocessed T1 and FLAIR images. These lesion masks were then transformed to ACPC space using linear registration. Only lesions >50mm^3^ were included in analysis.

For each subject, lesion masks for each pair of neighbouring time-points were then processed through our in-house software to determine the degree of chronic lesion expansion between consecutive time-points, while correcting for brain atrophy-related displacement of lesions, as described previously (Fig.1 bottom row).[1] The cumulative volume of lesion expansion for each patient was then computed by summating annual expansion values. To account for difference in duration of follow-up between subjects an average annual lesion expansion volume was used for analysis (Fig.1 bottom row, right panel).

A progressive volume/severity index (PVSI), as a measure of tissue damage in expanding part of chronic lesions was calculated by multiplying volume of lesion expansion by change of MD in the corresponding (expanding) area, as described previously.[3] Progressive tissue destruction inside chronic lesions was measured as an average annual increase of MD using the baseline lesion mask, which was adjusted to correct for brain atrophy-related displacement of lesions at follow-up, as described previously.[2]

**Identification of new acute lesions.**

This study focused on the analysis of the relationship between CP enlargement and chronic and acute lesions. Additional steps were therefore undertaken to identify and differentiate acute lesions that occurred during the study period.

Gadolinium-enhancing lesions that were detected at any time point were considered “new lesions” and excluded from the analysis of chronic lesion expansion for the next annual interval. However, gadolinium enhancement within active MS lesions usually does not persist beyond 2 months, after which newly formed T2 hyperintense lesions continue to shrink in size for another 3-5 months, reflecting resolution of edema and, potentially, tissue repair including remyelination [4]. Therefore, to accurately identify recent acute lesions, we further examined all lesions markedly (> 20%) shrinking between consecutive time-points; and determined whether these lesions were newly formed at the previous time-point or already existing. (Suppl Fig.2) All newly formed lesions at the previous time point (Suppl Fig.2 b) were classified as 'acute lesions' and were only included in the chronic lesion analysis from the subsequent time-point onwards (Suppl Fig.2 d).

**Volumetric brain analysis.**

Volumetric measures were obtained using AssemblyNet, an AI brain segmentation tool,[5] on T1 images in ACPC space.

The following metrics were analysed: total brain atrophy, white matter atrophy, grey matter atrophy, cortex and deep grey matter atrophy and ventricular volume change. For this study, volumetric change of ventricles was used as a measure of central brain atrophy (CBA).[6] [7] (Fig.1 middle row)

The cerebellum was excluded from the white and grey matter analysis.

To account for variability in the number of follow-up years between subjects, we established “a volume change per year” metric as a measure of atrophy. This involved calculating the annual change for each MRI measure and generating a line of best fit, the gradient of which represents annual change (see for instance Fig. 1 middle row, right panel).

Refferences.

1. **Klistorner S, Barnett MH, Yiannikas C, *et al.*** Expansion of chronic lesions is linked to disease progression in relapsing–remitting multiple sclerosis patients. *Multiple Sclerosis Journal*. 2021; **27**(10):1533–1542.

2. **Klistorner A, Wang C, Yiannikas C, *et al.*** Evidence of progressive tissue loss in the core of chronic MS lesions: A longitudinal DTI study. *Neuroimage Clin*. 2018; **17**:1028–1035.

3. **Klistorner S**, **Barnett MH**, **Klistorner A**. Mechanisms of central brain atrophy in multiple sclerosis. *MSJ*. 2022; **28**:2038–2045.

4. **Rovira A**, **Auger C**, **Alonso J**. Magnetic resonance monitoring of lesion evolution in multiple sclerosis. *Ther Adv Neurol Disord*. 2013; **6**(5):298–310.

5. **Coupé P, Mansencal B, Clément M, *et al.*** AssemblyNet: A large ensemble of CNNs for 3D whole brain MRI segmentation. *Neuroimage*. 2020; **219**:117–126.

6. **Dwyer M, Silva D, Bergsland N, *et al.*** Neurological software tool for reliable atrophy measurement (NeuroSTREAM) of the lateral ventricles on clinical-quality T2-FLAIR MRI scans in multiple sclerosis. *Neuroimage Clin*. 2017; **ahead of p**.

7. **Kalkers N**, **Vrenken H**, **Uitdehaag B**, **Polman C**, **Barkhof F**. Brain atrophy in multiple sclerosis: impact of lesions and of damage of whole brain tissue. *Multiple Sclerosis*. 2002; **8**(5):410–414. Available at: http://msj.sagepub.com/cgi/doi/10.1191/1352458502ms833oa [Accessed August 29, 2014].

**Supplementary figure legends.**

**Supplementary Fig. 1.** Diagram demonstrating the workflow for MRI analysis. Note that lesion segmentation was performed using T1 and FLAIR images in native space, while all other analysis-in ACPC space.


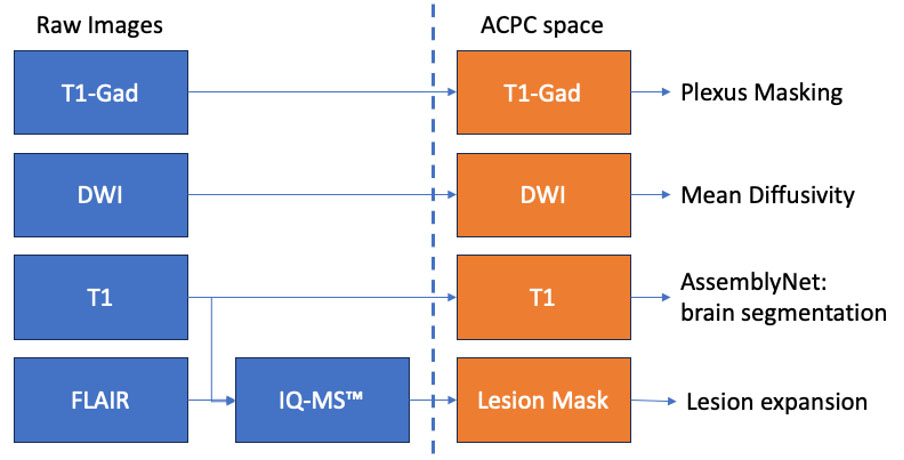


**Supplementary Fig. 2.** Example of new lesion identification. New lesion appeared at 12 m (b) and continued to shrink between 12 and 24 months. As a result, this lesion was only included in analysis of chronic lesions from 24 months onwards.


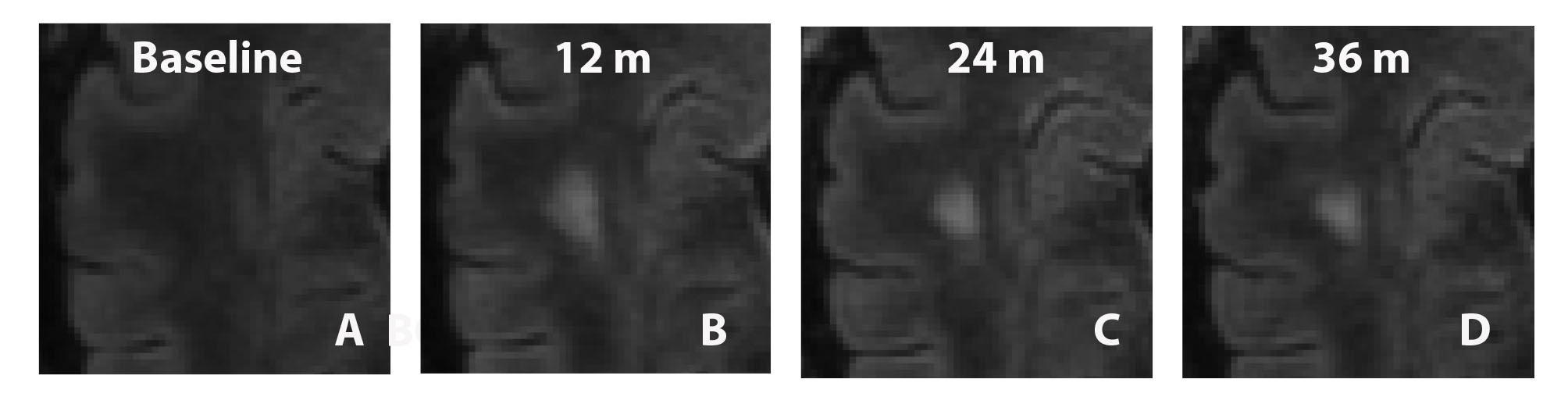

Supplement: sj-docx-1-msj-10.1177_13524585241228423 – Supplemental material for Longitudinal enlargement of choroid plexus is associated with chronic lesion expansion and neurodegeneration in RRMS patients [file sj-docx-1-msj-10.1177_13524585241228423.docx]
